# Autoimmune Polyglandular Syndrome Type 3 with Anorexia

**DOI:** 10.1155/2012/657156

**Published:** 2012-12-13

**Authors:** Toshio Kahara, Hitomi Wakakuri, Juri Takatsuji, Iori Motoo, Kosuke R. Shima, Kazuhide Ishikura, Rika Usuda, Yatsugi Noda

**Affiliations:** Department of Internal Medicine, Toyama Prefectural Central Hospital, 2-2-78 Nishinagae, Toyama 930-8550, Japan

## Abstract

A 71-year-old man with diabetes mellitus visited our hospital with complaints of anorexia and weight loss (12 kg/3 months). He had megaloblastic anemia, cobalamin level was low, and autoantibody to intrinsic factor was positive. He was treated with intramuscular cyanocobalamin, and he was able to consume meals. GAD autoantibody and ICA were positive, and he was diagnosed with slowly progressive type 1 diabetes mellitus (SPIDDM). Thyroid autoantibodies were positive. According to these findings, he was diagnosed with autoimmune polyglandular syndrome type 3 with SPIDDM, pernicious anemia, and Hashimoto's thyroiditis. Extended periods of cobalamin deficiency can cause serious complications such as ataxia and dementia, and these complications may not be reversible if replacement therapy with cobalamin is delayed. Although type 1 diabetes mellitus with coexisting pernicious anemia is very rare in Japan, physicians should consider the possibility of pernicious anemia when patients with diabetes mellitus have cryptogenic anorexia with the finding of significant macrocytosis (MCV > 100 fL).

## 1. Introduction 

Autoimmune polyglandular syndrome (APS) was described by Neufeld et al. in 1980 as an autoimmune disease that involves multiorgan failure [1]. APS type 2 is known as Schmidt's syndrome and is defined by the presence of adrenal insufficiency and autoimmune thyroid disease. Type 1 diabetes mellitus, gonadal failure, pernicious anemia, and myasthenia gravis can also occur. On the other hand, APS type 3 is an autoimmune thyroid disease without adrenal insufficiency and another associated autoimmune disease such as type 1 diabetes mellitus, pernicious anemia, vitiligo, and/or alopecia. Although patients with adrenal insufficiency frequently complain of gastrointestinal symptoms such as poor appetite [2], patients with pernicious anemia can also have anorexia [3]. We describe a case of APS type 3 in a diabetic patient with anorexia.

## 2. Case Report 

A 71-year-old man visited our hospital with complaints of anorexia and weight loss (12 kg/3 months). In his family history, his parents and sister had diabetes mellitus. He was diagnosed with diabetes mellitus on a medical examination at the age of 63, and he had been treated with 3 mg of glimepiride and 15 mg of pioglitazone in a neighboring hospital. A physical examination on admission revealed the following: height, 168 cm; body weight, 63 kg; body mass index (BMI), 22.3 kg/m^2^; blood pressure, 126/72 mmHg; body temperature, 36.1°C; consciousness, alert; skin, no pigmentation, no vitiligo, no alopecia; palpebral conjunctiva, anemic; oral cavity, smooth tongue, no pigmentation; neck, diffuse goiter; and ankle vibratory sensation, 3 seconds (right)/4 seconds (left). No abnormal findings were found on chest or abdominal examinations. Pathological reflexes and paralysis were not evident.

As is shown in Table 1, he had megaloblastic anemia (RBC 211 × 10^6^/*μ*L, Hb 9.3 g/dL, Ht 26.5%, MCV 125.6 fL, MCH 44.1 pg, and MCHC 35.1%). Cobalamin (vitamin B12) level was low (66 pg/mL), and autoantibody to intrinsic factor was positive. Gastroscopy revealed atrophic gastritis. These findings were consistent with pernicious anemia. He was treated with intramuscular cyanocobalamin (500 *μ*g × 10 days), and he was able to consume meals. His body weight recovered to 69 kg three months later. He visited our hospital with complaints of anorexia, but adrenal insufficiency was absent (urinary excretion of cortisol 150 *μ*g/day).

His insulin secretory capacity was not impaired (urinary excretion of C-peptide 61.0 *μ*g/day), but antiglutamic acid decarboxylase autoantibody (GAD autoantibody 15,000 U/mL) and islet cell antibody (ICA) were positive. He was diagnosed with slowly progressive type 1 diabetes mellitus (SPIDDM). The findings of diabetic retinopathy were absent, but his urinary excretion of albumin was 63.0 mg/g-Cr. He also noticed numbness of the foot, and vibratory sensation of his ankle was reduced. His blood glucose level worsened after recovery of appetite. HbA1c (NGSP) became 9.9% during three months, and he was treated with multiple daily injections of insulin (total: 28 units per day). Thyroid function was within normal limits, but thyroid autoantibodies were positive (antithyroid peroxidase antibody 21.1 U/mL, antithyroglobulin antibody 2.3 U/mL). According to these findings, he was diagnosed with APS type 3 with SPIDDM, pernicious anemia, and Hashimoto's thyroiditis. HLA haplotype revealed DRB1 *0401-DQB1 *0301, DRB1 *1302-DQB1 *0604, which is not an HLA haplotype susceptible to SPIDDM [4].

## 3. Discussion 

The prevalence of pernicious anemia is 0.15–1.0% in the general population, the prevalence increases with age, and the prevalence is increased by 3- to 5-fold in patients with autoimmune thyroid disease or type 1 diabetes mellitus [5]. In a long-term follow-up study in patients with APS in Germany, type 1 diabetes mellitus, Hashimoto's thyroiditis, Addison's disease, and pernicious anemia were observed in 61%, 33%, 19%, and 5% of patients, respectively [6]. On the other hand, Hibi et al. studied 1572 Japanese type 1 diabetes mellitus, thirty-three patients had autoimmune thyroid disease (2.10%) and only one patient had pernicious anemia [7]. Kinoshita et al. reported that type 1 diabetes mellitus with coexisting pernicious anemia was approximately 8 cases in Japan [8]. 

Pernicious anemia is an organ-specific autoimmune disease, and autoantibody to intrinsic factor is found in the circulation and in gastric secretions. Pernicious anemia accounts for 15–25% of cobalamin deficiency [3]. Cobalamin is required for DNA synthesis, and the major organs affected by cobalamin deficiency are those in which cell turnover is rapid, such as the bone marrow and the gastrointestinal tract. The patient sometimes complains of a sore tongue, which on inspection will be smooth and beefy red (atrophic glossitis). Patients with pernicious anemia have delayed gastric emptying [9], and appetite loss also may be evident. Intramuscular cyanocobalamin is an effective treatment in these patients. 

Cobalamin deficiency may cause peripheral neuropathy, such as numbness, as well as diabetic neuropathy. Extended periods of cobalamin deficiency can cause serious complications such as ataxia and dementia, and these complications may not be reversible if replacement therapy with cobalamin is delayed. Physicians should consider the possibility of pernicious anemia when patients with diabetes mellitus have cryptogenic anorexia with the finding of significant macrocytosis (MCV > 100 fL). 

## Figures and Tables

**Table 1:** Laboratory data on admission.

		Reference range
Cell blood count		
White blood cells	2.5 × 10^3^/*μ*L	4.0–9.0 × 10^3^
Neutrophils	56.2%	34.6–71.4
Eosinophils	0.4%	0–7.8
Lymphocytes	39.8%	19.6–52.7
Monocytes	3.6%	2.4–11.8
Red blood cells	2.11 × 10^6^/*μ*L	4.1–5.3 × 10^6^
Hemoglobin	9.3 g/dL	14.0–18.0
Hematocrit	26.5%	35.0–48.0
Mean corpuscular volume	125.6 fL	85.0–97.0
Mean corpuscular hemoglobin	44.1 pg	28.0–34.0
Mean corpuscular hemoglobin concentration	35.1%	32.0–36.0
Platelets	289 × 10^3^/*μ*L	150–350 × 10^3^
Reticulocytes	29.7 × 10^3^/*μ*L	36.0–104.0 × 10^3^
Blood chemistry		
Total protein	6.8 g/dL	6.7–8.3
Aspartate aminotransferase	44 IU/L	12–31
Alanine aminotransferase	41 IU/L	8–40
Alkaline phosphatase	240 IU/L	100–330
Lactate dehydrogenase	719 IU/L	110–210
*γ*-Glutamyl transpeptidase	27 IU/L	9–49
Creatine kinase	23 IU/L	65–275
Total bilirubin	3.2 mg/dL	0.2–1.2
Indirect bilirubin	3 mg/dL	0–0.8
Total cholesterol	107 mg/dL	150–220
Triglyceride	124 mg/dL	30–150
Blood urea nitrogen	20 mg/dL	8–22
Creatinine	0.6 mg/dL	0.6–1.1
Uric acid	3.3 mg/dL	3.0–7.0
Sodium	135 mEq/L	138–146
Potassium	4.3 mEq/L	3.6–4.9
Chloride	101 mEq/L	99–109
Calcium	9.1 mg/dL	8.7–11.0
Phosphorus	3.4 mg/dL	2.5–4.5
Iron	196 *μ*g/dL	54–181
Ferritin	220.5 ng/mL	21.8–274.7
Haptoglobin	1.0 mg/dL	19–170
Cobalamin	66 pg/mL	233–914
Folic acid	19.4 ng/mL	3.6–12.9
Antinuclear antibody	×80 (Homogeneous, speckled)	
Anti-intrinsic factor autoantibody	(+)	
Diabetes Mellitus		
Fasting plasma glucose	126 mg/dL	70–109
Hemoglobin A1c (NGSP)	6.6%	4.6–6.2
C-peptide	2.0 ng/mL	0.8–2.5
Urinary excretion of C-peptide	61.0 *μ*g/day	22.8–155.2
Urinary excretion of albumin	63.0 mg/g-Cr	0–24.6
GAD autoantibody	15,000 U/mL	0–1.4
IA-2 antibody	<0.4 U/mL	0–0.4
Islet cell antibody	(+)	
Thyroid hormone		
Thyrotropin	0.81 *μ*IU/mL	0.35–4.94
Free triiodothyronine	3.0 pg/mL	1.7–3.7
Free thyroxine	1.2 ng/dL	0.7–1.5
Thyroglobulin	2.9 ng/mL	0–32.7
Antithyroglobulin antibody	2.3 U/mL	<0.3
Antithyroid peroxidase antibody	21.1 U/mL	<0.3
Thyrotropin receptor antibody	<1.0 IU/L	<1.0
Adrenal hormone		
Adrenocorticotropic hormone	16.2 pg/mL	7.2–63.3
Cortisol	10.0 *μ*g/dL	4.5–21.1
Urinary excretion of cortisol	150.0 *μ*g/day	26.0–187.0
Antiadrenal autoantibodies	(−)	
Antipituitary autoantibodies	(−)	
